# The Klotho gene G-395A polymorphism and metabolic syndrome in very elderly people

**DOI:** 10.1186/s12877-016-0221-6

**Published:** 2016-02-15

**Authors:** Li Luo, Qiukui Hao, Birong Dong, Ming Yang

**Affiliations:** The Center of Gerontology and Geriatrics, West China Hospital, Sichuan University, No.37 Guoxue Lane, Chengdu, Sichuan China

**Keywords:** Klotho gene, Single nucleotide polymorphism, Metabolic syndrome, Nonagenarian, Centenarian

## Abstract

**Background:**

To investigate the possible association of the Klotho G-395A polymorphism and metabolic syndrome (MetS) among a population of Chinese nonagenarians and centenarians.

**Methods:**

Subjects were from the Project of Longevity and Aging in Dujiangyan (PLAD). The genotyping of G-395A (rs1207568) in the promoter region of the Klotho gene was performed using the TaqMan allelic discrimination assay. MetS was diagnosed according to the International Diabetes Federation criteria.

**Results:**

We included 695 subjects aged 93.5 ± 3.2 years. G and A allele frequencies were 0.852 and 0.148, respectively. In the whole population, the frequency of MetS was 10.8 % and 5.9 % in the GG and GA + AA genotype group, respectively (*p* = 0.004). The -395A allele carriers had significantly lower risk of MetS in the whole population (odd ratio [OR] 0.50, 95 % confidential interval [CI] 0.25 to 0.98) and in women (OR 0.51, 95 % CI 0.24 to 0.97), but not in men (OR 0.42, 95 % CI 0.05 to 3.85). In the whole population and women, the relationship between the Klotho G-395A SNP and MetS might due to its influence on high blood pressure (OR 0.48, 95 % CI 0.34 to 0.67; OR 0.47, 95 % CI 0.31 to 0.71, respectively) and hypertriglyceridemia (OR 0.66, 95 % CI 0.39 to 0.95; OR 0.54, 95 % CI 0.31 to 0.98, respectively). In men, this relationship might due to its influence on high blood pressure (OR 0.47, 95 % CI 0.25 to 0.90) and low HDL-C (OR 0.69, 95 % CI 0.27 to 0.93).

**Conclusions:**

The -395A allele carriers of the Klotho gene were correlated with lower risk of MetS among Chinese nonagenarians and centenarians, especially in women.

**Electronic supplementary material:**

The online version of this article (doi:10.1186/s12877-016-0221-6) contains supplementary material, which is available to authorized users.

## Background

Metabolic syndrome (MetS), a cluster of cardiometabolic risk factors including abdominal obesity, hyperglycemia, dyslipidemia and hypertension, increases with aging [1]. In elderly adults, MetS not only increases the risk of cardiovascular diseases and type 2 diabetes [2], but also is associated with cognitive decline [3] and disability [1]. Current evidence suggests that MetS is partly heritable, and genetic factors plays a greater role than environment factors on the incidence of MetS [4, 5]. Single nucleotide polymorphism (SNP) is one of the most active research fields of genetic researches. A recent systematic review found that eight SNPs in six genes (GNB3, TCF7L2, APOA5, APOC3, APOE, and CETP) were associated with MetS [6].

Klotho gene is an aging associated gene discovered by Kuro-o et al. during the development of hypertensive transgenic mice models [7]. Since then, more than 10 SNPs in the human Klotho gene have been identified [8], among which, the G-395A (rs1207568) SNP in the promoter region of Klotho gene was related to hypertension [9] or high systolic blood pressure ^10^ in previous studies [10] in previous studies. In addition, Shimoyama and colleagues found that the Klotho G-395A SNP was related to lipid metabolism in men and glucose metabolism in women among Japanese health subjects [11]. The same research team also found that the Klotho G-395A SNP was associated with low levels of low-density lipoprotein cholesterol (LDL-C) in Japanese hemodialysis patients [12].

Based on these findings, we speculated that the Klotho G-395A SNP may be a candidate SNP related to MetS. In 2005, we performed a cross-sectional study [13, 14] including 870 Chinese adults aged 90 years and older, and built a relevant DNA specimen database. Most of these participants lived in their homeland during their entire lifetime and were never exposed to immigrants, which can represent Chinese well. It offered us an opportunity to investigate the genetic factors of MetS in this special population. In this study, we aimed to examine the possible association between the Klotho G-395A SNP and MetS among this long-lived population.

## Methods

### Study subjects

Subjects were from the Project of Longevity and Aging in Dujiangyan (PLAD) [13, 14], a cross-sectional study conducted in the rural area of Dujiangyan, a small town in southwest China, in April 2005. This study was conducted in order to investigate the relationship between environment, lifestyle and age-related diseases, and longevity. A total of 1115 adults aged 90 years and older dwelling in this area were screened, among which, 870 subjects (men: 280 cases; women: 590 cases) agreed to participant. Trained staff visited all study participants at their homes or at community centers. Face-to-face interviews were performed for data collection. Anthropometric measurements were performed and biological specimens were collected by trained staff. Subjects without DNA samples (160 cases) or the results of waist circumference (4 cases) and high density lipoprotein cholesterol (HDL-C, 11 cases) were excluded from the current analyses. As a result, the study population ultimately consisted of 695 subjects (men: 220 cases; women: 475 cases). The Research Ethics Committee of Sichuan University approved the study protocol. Written informed consent was obtained from all of the participants or their legal proxies.

### Genotyping of the Klotho gene G-395A polymorphism

Genomic DNA was isolated from the whole blood samples, drawn from antecubital vein, using commercial DNA isolation kits from QIAGEN (Chatsworth, CA, USA), according to standard procedures. The genotyping of G-395A (rs1207568) in the promoter region of the Klotho gene was performed using the TaqMan allelic discrimination assay as previously reported by Wang et al. [9].

The following primers and probes (Takara, Dalian, China) were used:

- Forward primer 5′-TAGGGCCCGGCAGGAT-3′;
- Reverse primer 5’-CCTGGAGCGGCTTCGTC-3’;
- Probe A 5’-(FAM) CCCCAAGTCGGGAAAAGTTGGTC (TAMRA)-3’;
- Probe G 5’-(HEX) CCCCCAAGTCGGGGAAAGTTGGTC (TAMRA)-3’.

The PCR reaction was performed in 20 μl reaction volumes containing 10 μl of Premix Ex Taq, 1.5 μl of each primer, 0.5 μl of probe A, 1 μl of probe G, 1 μl of genomic DNA, and 4.25 μl of sterile double distilled water. The reaction conditions were as follows: an initial denaturation step at 95 °C for 30 s, followed by 40 cycles of denaturation at 95 °C for 5 s, and annealing for 30 s at 60 °C. The PCR reaction was performed and analyzed on a Thermal Cycler Dice Real Time System (Takara, Dalian, China).

In addition, we randomly selected 10 % of the samples for sequencing in both the forward and reverse directions to confirm the genotypes of G-395A in the promoter of Klotho gene. The results of the sequencing were identical to the results of the TaqMan allelic discrimination assay.

### Diagnosis of MetS

MetS was diagnosed according to the International Diabetes Federation (IDF) criteria [15]: abdominal obesity (for Chinese people, waist circumference [WC] ≥ 90 cm in men and ≥ 80 cm in women), combined with any two or more of the following four factors: 1) fasting plasma glucose (FPG) ≥ 5.6 mmol/L or previously diagnosed type 2 diabetes; 2) systolic blood pressure (SBP) ≥ 130 mm Hg and/or diastolic blood pressure (DBP) ≥ 85 mm Hg or currently use of anti-hypertensive agents; 3) HDL-C < 1.29 mmol/L in women and <1.03 mmol/L in men or specific treatment for this lipid abnormality; 4) triglyceride (TG) ≥ 1.7 mmol/L or specific treatment for this lipid abnormality.

### Measurement of covariates

Body height and weight were measured using a wall-mounted stadiometer and a digital floor scale to the nearest 0.1 cm and 0.1 kg, respectively. Body mass index (BMI) was calculated by dividing body weight in kilograms by height in squared meters (kg/m^2^). In addition, the following information were included in the analyses: age, gender, and cigarette smoking habits, alcoholic habits, and exercise habits. Venous blood samples were collected after an overnight fast to measure the following biochemistry indicators: FPG, total cholesterol (TC), TG, HDL-C, LDL-C, and serum uric acid (SUA).

### Statistical analysis

All of the statistical analyses for this study were performed with SPSS for Windows software package, version 11.5 (SPSS Inc., Chicago, Illinois, USA). A two-tailed P <0.05 was considered statistically significant. The categorical variables are presented as percentage, whereas the continuous data are presented as the mean ± SD. A Chi-square test was performed to evaluate the allelic and genotypic frequencies that were calculated from the observed genotypic counts. Using Chi-square tests for categorical variables and one-way ANOVA for continuous variables, characteristics of subjects were compared between those with or without MetS and between those with or without the -395A allele, respectively. Because the prevalence of MetS was generally higher in women than in men [16], we reported the results in men and women, respectively, as well as in the whole population. In addition, odds ratios (OR) and 95 % confidential intervals (CI) for MetS were determined using binary logistic regression models.

## Results

### Clinical characteristics of study subjects

Among the 695 subjects for the current analyses, the mean age was 93.5 ± 3.2 years (range 90–108 years), 475 (68.3 %) were women. MetS was presented in 9.4 % of the whole population and it was more prevalent in women (12.2 %) than men (3.2 %) (*p* < 0.001). There was a significant difference in the distribution of many clinical parameters between subjects with or without MetS (Table 1). There were more women in the MetS group than in the non-MetS group (89.2 % vs. 66.2 %, *p* < 0.001). The mean values of WC, SBP, DBP, FPG, TG, LDL-C, SUA, and BMI were significantly higher in subjects with MetS compared to those without MetS, whereas the mean value of HDL-C was significantly lower in the MetS group. However, no significant difference was found between the two groups with respect to the mean value of TC.

**Table 1:** Characteristics of subjects according to MetS (*N* = 695)

Characteristics	Total (*N* = 695)			Men (*N* = 220)			Women (*N* = 475)		
	MetS (*N* = 65)	Non-MetS (*N* = 630)	*P*	MetS (*N* = 7)	Non-MetS (*N* = 213)	*P*	MetS (*N* = 58)	Non-MetS (*N* = 417)	*P*
Age (years)	93.3 ± 3.0	93.6 ± 3.2	0.532	92.7 ± 2.8	93.3 ± 3.1	0.606	93.4 ± 3.1	93.7 ± 3.3	0.505
Women (%)	89.2	66.2	<0.001	-	-	-	-	-	-
BMI (kg/m^2^)	22.3 ± 3.3	18.9 ± 3.6	<0.001	23.8 ± 4.5	19.6 ± 3.8	0.004	22.2 ± 3.1	18.6 ± 3.4	<0.001
WC (cm)	87.8 ± 5.2	76.5 ± 7.9	<0.001	92.1 ± 2.5	78.3 ± 7.3	<0.001	87.2 ± 5.2	75.6 ± 8.1	<0.001
SBP (mm Hg)	156 ± 18.2	138 ± 23.3	<0.001	152.9 ± 11.1	137.6 ± 20.3	0.049	156.8 ± 18.9	139.3 ± 24.7	<0.001
DBP (mm Hg)	78.4 ± 15.1	72.0 ± 11.6	<0.001	77.1 ± 7.6	73.1 ± 11.2	0.345	78.5 ± 15.8	71.4 ± 11.7	0.001
FPG (mmol/L)	5.1 ± 2.1	4.4 ± 1.3	<0.001	7.4 ± 2.8	4.5 ± 1.5	<0.001	4.9 ± 1.8	4.3 ± 1.2	0.022
TC (mom/L)	4.5 ± 1.0	4.6 ± 2.0	0.930	4.1 ± 1.1	5.3 ± 2.6	0.874	4.5 ± 1.0	4.2 ± 0.8	<0.001
TG (mmol/L)	2.0 ± 1.1	1.2 ± 0.6	<0.001	1.9 ± 1.0	1.0 ± 0.4	<0.001	2.1 ± 1.2	1.2 ± 0.6	<0.001
LDL-C (mmol/L)	2.6 ± 0.7	2.2 ± 0.6	<0.001	2.5 ± 0.7	2.1 ± 0.5	0.079	2.6 ± 0.7	2.3 ± 0.6	<0.001
HDL-C (mmol/L)	1.4 ± 0.5	1.6 ± 0.8	0.020	1.2 ± 0.3	1.5 ± 0.7	0.179	1.4 ± 0.5	1.7 ± 0.8	0.019
SUA (mmol/L)	342.4 ± 83.4	316.3 ± 88.3	0.023	394.7 ± 31.4	350.9 ± 87.6	0.189	336.0 ± 85.6	298.6 ± 83.4	0.002
Smoking habits* (%)	35.9	43.6	0.237	85.7	69.7	0.361	29.8	30.4	0.934
Alcohol habits** (%)	14.1	27.2	0.022	28.6	39.0	0.576	12.3	21.3	0.113
Exercise habits*** (%)	36.9	41.0	0.524	14.3	42.3	0.139	39.7	40.3	0.920
Klotho G-395A Genotypes									
GG (%)	81.5	69.2	0.046	85.7	72.8	0.447	81.0	67.4	0.034
GA (%)	18.5	28.9		14.3	27.2		19.0	29.7	
AA (%)	0	1.9		0	0		0	2.9	

*Abbreviations*: *BMI* body mass index, *DBP* diastolic blood pressure, *FPG* fasting plasma glucose, *HDL-C* high density lipoprotein cholesterol, *LDL-C* low density lipoprotein cholesterol, *MetS* metabolic syndrome, *SBP* systolic blood pressure, *SUA* serum uric acid, *TC* total cholesterol, *TG* triglyceride, *WC* waist circumference

*Indicates those who were a smoker at the time of the interview

**Indicates those who still drank alcohol at least once a day at the time of the interview

***Indicates those who still did some physical exercise at least 3 time per week at the time of the interview

Subgroup analysis showed similar results in women, except that the mean value of TG were significantly higher in women with MetS compared with those without MetS. In men, subjects with MetS had significantly higher mean values of BMI, WC, SBP, FPG, and TG compared with those without MetS, but no significant difference was observed between the MetS and non-MetS groups in terms of the mean values of DBP, TC, H DL-C, LDL-C, and SUA (Table 1).

### Incidence of the Klotho G-395A SNP

In the whole population, the genotype frequencies of the Klotho G-395A SNP were 70.4 % for GG genotype (*n* = 489), 29.5 % for GA genotype (*n* = 194), and 1.7 % for AA genotype (*n* = 12). These frequencies were in compliance with the Hardy-Weinberg equilibrium (*X*^2^ = 2.14; *p* = 0.14). G and A allele frequencies were 0.852 and 0.148, respectively. Incidence of each genotype of the Klotho G-395A SNP according to MetS and gender is shown in Table 1. The distribution of the Klotho G-395A genotypes was significantly different between the MetS and non-MetS groups in the whole population and in women, but not in men.

### The Klotho G-395A SNP and MetS

The characteristics of subjects according to the Klotho G-395A SNP was shown in Table 2. In the whole population, the frequency of MetS was 10.8 % in the GG genotype group and 5.9 % in the GA + AA genotype group (*p* = 0.004), and the result of logistic regression indicated that the -395A allele carriers had significantly lower risk of MetS after adjusting for age, gender, smoking habits, alcohol habits, exercise habits, BMI, and SUA (OR 0.50, 95 % CI 0.25 to 0.98) (Table 3).

**Table 2:** Characteristics of subjects according to the klotho G-395A polymorphism (*N* = 695)

Characteristics	Total (*N* = 695)			Men (*N* = 220)			Women (*N* = 475)		
	GG genotype (*N* = 490)	GA + AA genotype (*N* = 205)	*P*	GG genotype (*N* = 161)	GA + AA genotype (*N* = 59)	*P*	GG genotype (*N* = 329)	GA + AA genotype (*N* = 146)	*P*
Age (years)	93.6 ± 3.3	93.2 ± 2.9	0.074	93.4 ± 3.2	93.2 ± 3.0	0.679	93.8 ± 3.4	93.2 ± 2.9	0.073
Women (%)	67.1	71.2	0.292	-	-	-	-	-	-
BMI (kg/m^2^)	19.2 ± 3.8	19.4 ± 3.3	0.727	19.6 ± 4.1	20.0 ± 3.1	0.505	19.1 ± 3.6	19.1 ± 3.4	0.952
Smoking habits*	45.0	37.9	0.089	71.2	67.2	0.568	32.1	26.2	0.198
Alcohol habits**	25.5	27.2	0.645	42.1	29.3	0.086	17.4	26.4	0.026
Exercise habits***	41.2	39.3	0.652	42.0	39.7	0.753	40.7	39.2	0.748
SUA (mmol/L)	318.8 ± 87.5	318.7 ± 89.9	0.998	346.1 ± 81.3	369.3 ± 98.6	0.079	305.5 ± 87.4	298.2 ± 77.5	0.387
MetS (%)	10.8	5.9	0.040	3.7	1.7	0.447	14.3	7.5	0.038
Components of MetS									
Abdominal obesity (%)	25.5	26.3	0.819	9.3	5.1	0.310	33.4	34.9	0.750
Hypertriglyceridemia (%)	19.2	11.7	0.017	10.2	5.6	0.233	20.7	12.3	0.029
Low HDL-C (%)	16.1	14.1	0.623	9.9	1.7	0.043	19.2	19.7	0.894
High BP (%)	66.3	49.8	<0.001	62.1	45.8	0.030	68.4	51.4	<0.001
High FPG (%)	13.7	17.6	0.188	20.5	22.0	0.804	10.3	15.8	0.094

*Abbreviations*: *BMI* body mass index, *BP* blood pressure, *FPG* fasting plasma glucose, *HDL-C* high density lipoprotein cholesterol, *MetS* metabolic syndrome, *SUA* serum uric acid

*Indicates those who were a smoker at the time of the interview

**Indicates those who still drank alcohol at least once a day at the time of the interview

***Indicates those who still did some physical exercise at least 3 time per week at the time of the interview

**Table 3:** Estimate of the effect of klotho G-392A polymorphism on MetS and its components with logistic regression

	MetS	Components of MetS				
		Abdominal obesity	Hypertriglyceridemia	Low HDL-C	High BP	High FPG
Total						
GG genotype, OR^a^ (95 % CI)	1 (reference)	1 (reference)	1 (reference)	1 (reference)	1 (reference)	1 (reference)
GA + AA genotype, OR^a^ (95 % CI)	0.50 (0.25, 0.98)	0.95 (0.60, 1.49)	0.66 (0.39, 0.95)	0.84 (0.51, 1.37)	0.48 (0.34, 0.67)	1.47 (0.93, 2.31)
Men						
GG genotype, OR^b^ (95 % CI)	1 (reference)	1 (reference)	1 (reference)	1 (reference)	1 (reference)	1 (reference)
GA + AA genotype, OR^b^ (95 % CI)	0.42 (0.05, 3.85)	0.36 (0.16, 1.89)	1.66 (0.47, 5.80)	0.69 (0.27, 0.93)	0.47 (0.25, 0.90)	1.00 (0.47, 2.12)
Women						
GG genotype, OR^b^ (95 % CI)	1 (reference)	1 (reference)	1 (reference)	1 (reference)	1 (reference)	1 (reference)
GA + AA genotype, OR^b^ (95 % CI)	0.51 (0.24, 0.97)	1.06 (0.66, 1.71)	0.54 (0.31, 0.98)	0.99 (0.58, 1.68)	0.47 (0.31, 0.71)	1.93 (0.96, 3.51)

*Abbreviations*: *BMI* body mass index, *BP* blood pressure, *FPG* fasting plasma glucose, *HDL-C* high density lipoprotein cholesterol, *MetS* metabolic syndrome, *SUA* serum uric acid

^a^Adjusted for age, gender, smoking habits, alcohol habits, exercise habits, BMI, and SUA

^b^Adjusted for age, smoking habits, alcohol habits, exercise habits, BMI and SUA

In women, the frequencies of MetS were 14.3 % and 7.5 % in the GG and GA + AA groups, respectively (*p* = 0.038). The result of logistic regression showed that the -395A allele carriers had significantly lower risk of MetS after adjusting for relevant confounders (OR 0.51, 95 % CI 0.24 to 0.97) (Table 3).

In men, the frequencies of MetS were 3.7 % and 1.7 % in the GG and GA + AA groups, but the difference was not statistically significant (*p* = 0.447). Logistic regression analysis failed to find a significantly association between the Klotho G-395A SNP and MetS adjusting for relevant confounders (OR 0.42, 95 % CI 0.05 to 3.85) (Table 3).

### The Klotho G-395A SNP and components of MetS

In the whole population, the -395A allele carriers, compared with the control subjects, had significantly lower frequencies of high blood pressure (49.8 % vs. 66.3 %, *p* < 0.001) and hypertriglyceridemia (11.7 % vs. 19.2 %, *p* = 0.017). There was no significant association of the Klotho G-395A SNP with abdominal obesity, Low HDL-C, and high FPG (Table 2). After adjusting for relevant confounders, logistic regression analysis revealed that the Klotho G-395A SNP was significantly associated with high blood pressure (OR 0.48, 95 % CI 0.34 to 0.67) and hypertriglyceridemia (OR 0.66, 95 % CI 0.39 to 0.95) (Table 3).

In women, the −395 allele carriers also had significantly lower frequencies of high blood pressure (51.4 % vs 68.4 %, *p* < 0.001) and hypertriglyceridemia (12.3 % vs. 20.7, *p* = 0.029). In addition, the -395A allele carriers had a trend of higher frequency of high FPG, but it was not statistically significant (15.8 % vs. 10.3 %, *p* = 0.094). After adjusting for relevant confounders, logistic regression analysis showed that the Klotho G-395A SNP was significantly associated with high blood pressure (OR 0.47, 95 % CI 0.31 to 0.71) and hypertriglyceridemia (OR 0.54, 95 % CI 0.31 to 0.98) (Table 3).

In men, the −395 allele carriers had significantly lower frequencies of high blood pressure (45.8 % vs 62.1 %, *p* = 0.030) and low HDL-C (1.7 % vs. 9.9 %). After adjusting for relevant confounders, logistic regression analysis showed that the Klotho G-395A SNP was significantly associated with high blood pressure (OR 0.47, 95 % CI 0.25 to 0.90) and low HDL-C (OR 0.69, 95 % CI 0.27 to 0.93) (Table 3).

## Discussion

To the best of our knowledge, this is the first study to investigate the relationship between the Klotho G-395A SNP and MetS. The results of this study indicated that the -395A allele carriers of the Klotho gene were related to lower risk of MetS among Chinese nonagenarians and centenarians, especially in women. The association of the Klotho G-395A SNP with MetS could be linked to its observed influence on high blood pressure in both men and women, and hypertriglyceridemia in women. These findings were supported by previous studies which demonstrated that Klotho gene had an impact on multiple aspects of MetS including blood pressure, lipid metabolism and insulin resistance in different populations [11, 12, 17].

The association of the Klotho variant with the metabolic syndrome could be linked to its observed influence on high blood glucose, high blood pressure, insulin resistance, hypertriglyceridemia and endothelial function [17, 18]. The potential involvement of SNP G-395A in KLOTHO gene function and pathogenesis of the metabolic syndrome and these components remains speculative. Previous studies reported G-395A can influence the affinity of binding transcription factors, G-395A has strong Linkage Disequilibrium with other SNP and other gene (PPAR-γ), which could also act as a surrogate for other functional variant(s) nearby (such as G110C, C1818T and C2298T) [9, 17, 19, 20]. Furthermore, a previous study indicated that the G-395A SNP in the promoter region of Klotho gene might influence the function of Klotho gene by upregulating its expression and the Klotho protein may function as a humoral factor [9]. Thus, we speculated that the up-regulation of Klotho expression and functional link with other gene could also explain the observed association between the G-395A and the metabolic syndrome.

The minor allele frequency (MAF), the frequency of the -395A allele in this study, was 0.148. In previous studies, the MAF of the Klotho G-395A SNP were 0.191 in a Chinese Han population [9], from 0.155 to 0.171 in Korean populations [10] [21], from 0.128 to 0.168 in Japanese populations [11, 19], and 0.277 in a Turkish population [22]. Our finding was similar with the results of those studies conducted in East Asian populations, but inconsistent with the Turkish study [22]. One possible reason was the MAF of the Klotho G-395A SNP varied across ethnic populations. In addition, we did not identify the AA genotype in our population, whereas it varied from 1.7 % to 5.9 % in previous studies [10, 11, 21, 22]. The survival effect might account for this result as only nonagenarians and centenarians were included in this study.

In our study, the frequency of high blood pressure (defined as SBP ≥ 130 mm Hg and/or DBP ≥ 85 mm Hg) was significantly lower in the -395A allele carriers than the non-carriers among both men and women. Another Chinese study found similar results in women but not in men [9]. On the contrary, Rhee and colleague found that the mean SBP was significantly higher in the -395A allele carriers in health Korean women [10]. In addition, Shimoyama and colleagues reported no significant difference in the mean SBP between the -395A carriers and the non-carriers in healthy Japanese subjects [11]. Putting all these findings together, we may speculate that the association between blood pressure and the Klotho G-395A SNP varies in different ethnic populations.

The reason of the gender difference in the association of the Klotho G-395A SNP with lipid metabolism in this study remains unclear. Shimoyama et al. also found gender difference in the association between the Klotho G-395A SNP and lipid metabolism in Japanese people. The possible reason may be the gender-based difference in free radical homeostasis maintenance [23]. The Klotho gene has been reported to reduce oxidative stress by inhibiting the insulin/IGF-1 signaling pathway [8], whereas oxidative stress appears to be related to lipid metabolism [24].

It is notable that the prevalence of MetS in our study (9.4 %) was lower than that reported in other studies [1, 2]. In most developed countries the prevalence of MetS is approximately 20 % in the overall population, and increases with advancing age [1]. In China, a recent study reported that the prevalence of MetS was 18.4 % in adults according to the IDF criteria [25]. One possible reason for the low prevalence of MetS in our population was that people with MetS were prone to multiple morbidities and therefore might have low probability to live to more than ninety. However, to our knowledge, there was no other study regarding the prevalence of MetS in long-lived populations. Therefore, we could not found more evidence to support this inference.

There is robust evidence that hyperuricemia is associated with MetS, and some researchers argue that hyperuricemia should be considered as a new marker for MetS [26]. In this study, we found that the mean value of uric acid was significantly higher in subjects with MetS compared with those without MetS in all and female subjects, but the difference was not significant in men. In addition, we did not find the association between uric acid and the Klotho G-395A SNP. However, Shimoyama et al. reported that uric acid was significantly high in the -395A allele carriers of the Klotho gene compared with non-carriers in Japanese hemodialysis patients [12].

This study has some limitations. First, there were 870 subjects in the PLAD study, however, 175 subjects were excluded in the current analyses because of missing essential data. The exclusion of participants might induce selection bias. Second, because women generally have a longer life expectancy than men, a significant gender imbalance existed in our study population. The relatively small sample size of men (220 cases) reduced the possibility for detecting the possible association of Klotho G-395A SNP with MetS and its components and some conclusion may biased by the small sample size. Third, we only included the very old people in this study (90 or older), there might be survival bias. However, this is inherent to a study of individuals of this age-group. In conclusion, the Klotho G-395A SNP is associated with MetS in very elderly people, especially in women. This association could be linked to its influence on blood pressure and lipid metabolism. Further cross-sectional and longitudinal studies with larger sample size are warranted to investigate this association in different ethnic populations.

## Conclusion

The results of this study indicates that the -395A allele carriers of the Klotho gene are correlated with lower risk of MetS in a sample of Chinese nonagenarians and centenarians, especially in women. This relationship needs to be further investigated in the future.

### Ethics approval and consent to participate

The study protocol was approved by the Research Ethics Committee of Sichuan University. Written informed consent was obtained from all of the participants (or their legal proxies).

### Consent for publication

Not applicable.

### Availability of data and materials

The anonymized genetic dataset supporting the conclusions of this article is included within the article and its Additional file 1. The other data in this study will not be shared because many information are from local government and medical records, we are not allowed to share these data due to the privacy policy.

## Additional file

Additional file 1:
**the SNP data of the study population.** (XLS 36 kb)

## Abbreviations

MetS: Metabolic syndrome
PLAD: the Project of Longevity and Aging in Dujiangyan
OR: Odd ratio
CI: Confidential interval
SNP: Single nucleotide polymorphism
LDL-C: Low density lipoprotein cholesterol
HDL-C: High density lipoprotein cholesterol
IDF: the International diabetes federation
WC: Waist circumference
FPG: Asting plasma glucose
SBP: Systolic blood pressure
DBP: Diastolic blood pressure
TG: Triglyceride
BMI: Body mass index
TC: Total cholesterol
SUA: Serum uric acid
MAF: The minor allele frequency
